# Assessment of Microalbuminuria for Early Diagnosis and Risk Prediction in Dengue Infections

**DOI:** 10.1371/journal.pone.0054538

**Published:** 2013-01-22

**Authors:** Nguyen Thi Hanh Tien, Phung Khanh Lam, Huynh Thi Le Duyen, Tran Van Ngoc, Phan Thi Thanh Ha, Nguyen Tan Thanh Kieu, Cameron Simmons, Marcel Wolbers, Bridget Wills

**Affiliations:** 1 Oxford University Clinical Research Unit, Hospital for Tropical Diseases, District 5, Ho Chi Minh City, Vietnam; 2 Centre for Tropical Medicine, Nuffield Department of Medicine, University of Oxford, Oxford, United Kingdom; 3 Hospital for Tropical Diseases, District 5, Ho Chi Minh City, Vietnam; 4 District 8 Hospital, District 8, Ho Chi Minh City, Vietnam; Duke-National University of Singapore Graduate Medical School, Singapore

## Abstract

**Background:**

Dengue is the most important arboviral infection of humans. Following an initial febrile period, a small proportion of infected patients develop a vasculopathy, with children at particular risk for severe vascular leakage and shock. Differentiation between dengue and other common childhood illnesses is difficult during the early febrile phase, and risk prediction for development of shock is poor. The presence of microalbuminuria is recognized as a useful early predictor for subsequent complications in a number of other disorders with vascular involvement. Significant proteinuria occurs in association with dengue shock syndrome and it is possible that early-phase microalbuminuria may be helpful both for diagnosis of dengue and for identification of patients likely to develop severe disease.

**Methodology/Principal Findings:**

We measured formal urine albumin to creatinine ratios (UACRs) in daily samples obtained from a large cohort of children with suspected dengue recruited at two outpatient clinics in Ho Chi Minh City, Vietnam. Although UACRs were increased in the 465 confirmed dengue patients, with a significant time trend showing peak values around the critical period for dengue-associated plasma leakage, urine albumin excretion was also increased in the comparison group of 391 patients with other febrile illnesses (OFI). The dengue patients generally had higher UACRs than the OFI patients, but microalbuminuria, using the conventional cutoff of 30 mg albumin/g creatinine discriminated poorly between the two diagnostic groups in the early febrile phase. Secondly UACRs did not prove useful in predicting either development of warning signs for severe dengue or need for hospitalization.

**Conclusion/Significance:**

Low-level albuminuria is common, even in relatively mild dengue infections, but is also present in many OFIs. Simple point-of-care UACR tests are unlikely to be useful for early diagnosis or risk prediction in dengue endemic areas.

## Introduction

Dengue is the most important arboviral infection affecting humans and represents a major global public health problem. Disease can be caused by any one of four dengue viral serotypes (DENV1-4) transmitted by Aedes mosquitoes. Following recent geographical expansion of the major mosquito vectors it is estimated that 2.5 billion people now live in areas of risk, and about 50 million infections occur annually [1].

Clinical presentation of dengue infection varies widely, ranging from mild febrile illness to severe and fatal disease. In a small proportion of cases, typically children and young adults, a characteristic vasculopathy develops, featuring increased systemic vascular permeability, coagulation abnormalities, and bleeding manifestations. Vascular leakage may be severe, especially in children, resulting in circulatory compromise and the potentially life-threatening dengue shock syndrome (DSS). However, typically, DSS does not occur until 4–5 days into the illness at a time when the host immune response is well established and viraemia and fever are resolving. Currently no effective antiviral agents are available and treatment remains supportive, with particular emphasis on careful fluid management [1].

Dengue shares many clinical features with other febrile illnesses (OFI) that are common in dengue-endemic regions; for example measles, typhoid, leptospirosis, and influenza can all be confused with dengue during the febrile phase [2], [3]. In addition, the risk factors for development of severe disease are poorly characterised and consequently uncomplicated cases are frequently hospitalised for observation during the critical phase for leakage. Thus as well as the financial and social cost to patients and their families, dengue imposes major demands on healthcare systems in endemic areas. Improvements in early diagnosis and risk prediction for severe disease are urgently needed, particularly with respect to identification of simple clinical and/or laboratory indicators that are practical and affordable for use in resource poor countries. A variety of features, including platelet count, white blood count, liver enzyme abnormalities, and presence of rash, that differentiate between dengue and OFI have been reported, but the cut-offs and combinations suggested in the various prognostic models proposed, differ considerably, and the diagnostic utility of such tests in routine clinical practice remains limited [2], [4]–[6]. Several warning signs for the development of severe dengue are also recommended in the WHO 2009 Guidelines, primarily based on expert opinion rather than formal evidence from clinical research studies [1].

Microalbuminuria has become accepted as a useful screening tool for early identification of complications in disorders with vascular involvement, including renal diseases, diabetes, and arteriosclerosis, and it is now established as an early correlate of all cause mortality in the general population [7]–[9]. Proteinuria is sometimes observed in dengue cases, typically without evidence of renal involvement [10], [11], and marked increases in fractional clearances of several endogenous proteins have been documented among children with DSS [12]. The presence and/or severity of microalbuminuria have been postulated as potential risk predictors for severe dengue, but there is little information on the magnitude, timing of onset, or evolution of urinary protein excretion during infection. Formal 24-hour urinary albumin measurements are cumbersome and time-consuming to perform, but measurement of urine albumin:creatinine ratios (UACRs) on random urine samples has become established as an acceptable alternative for screening large patient populations [13]. Simple point-of-care tests to quantify urinary albumin excretion are also now available, and could potentially provide useful information for clinical management of suspected dengue cases. We therefore set out to characterize the kinetics and magnitude of urinary albumin excretion in a group of Vietnamese children with suspected dengue, in order to assess whether microalbuminuria during the early febrile phase is helpful in differentiating dengue from OFI, or in risk prediction for the subsequent development of vascular leakage.

## Materials and Methods

### Patients and Clinical Methods

We conducted a prospective descriptive study of febrile children, aged 5–15 years, attending two primary health care clinics in Ho Chi Minh City, Vietnam. Clinic A is a single-handed practice run by a senior paediatrician, while Clinic B is the walk-in paediatric clinic at District 8 Hospital. This study forms one part of a large community study on dengue, the clinical aspects of which have been described previously, but briefly all children presenting with fever and clinically suspected dengue to either clinic were eligible for enrolment following written informed consent [14]. Recruitment was targeted towards patients presenting during the early febrile period, ideally within the first 72 hours from fever onset, although patients presenting up to 96 hours from fever onset could be enrolled. Patients were seen daily until afebrile for two consecutive days, with detailed clinical information recorded in a standard format and a 1 ml EDTA blood sample obtained for clinical (haematocrit estimation and platelet count) and diagnostic purposes, together with a random urine sample. Clinic physicians were responsible for all management decisions; if hospitalization was considered necessary the children were admitted to HTD and the daily assessments continued, following the same protocol as the outpatient subjects. Patients were invited to attend for review 2–4 weeks from illness onset.

Illness day 1 was defined as the day of reported fever onset. Defervescence day was defined as the first day with no history of fever since the previous day’s visit and with a measured temperature ≤37.5°C in the clinic. The following outcomes were summarised from the daily assessments: the platelet nadir between days 3–8 of illness; the presence or absence of skin and/or mucosal bleeding; the percentage hemoconcentration, defined as the percentage increase in haematocrit comparing the maximum value recorded between days 3–8 of illness, to a baseline value taken as the lowest result obtained on or before illness day 2 or after day 14, or a local population value matched for age and sex if no individual baseline was available [14].

### Ethics Statement

Ethical approvals were obtained from the Ethical Committees of the Hospital for Tropical Diseases (HTD) and of District 8 Hospital in Ho Chi Minh City, as well as the Oxford Tropical Research Ethics Committee. For all participants a parent/guardian gave written informed consent and children over 12 years gave assent.

### Dengue Diagnostics

Dengue diagnostic capture IgM and IgG ELISAs were performed on paired enrolment and convalescent specimens [15], together with detection of DENV RNA using RT-PCR on the enrolment specimen [16]. A laboratory-confirmed case was defined by detection of DENV RNA in plasma or by seroconversion in the capture IgM or IgG ELISA. Patients in whom all dengue diagnostics were conclusively negative (RT-PCR and paired serology), and for whom there was no evidence of another specific infectious disease were defined as having OFI. Patients with negative enrolment RT-PCR but indeterminate serology results, usually due to missing convalescent samples, were considered unclassifiable.

### Urine Albumin Quantification and Urine Albumin Creatinine Ratio

Urine albumin and creatinine measurements were performed on all enrolment and discharge urine samples from children with a final diagnosis of confirmed dengue or OFI. In addition serial daily analyses were carried out on specimens obtained from all patients attending Clinic A, and on patients with confirmed dengue from Clinic B. The daily samples obtained from OFI patients attending Clinic B were not analysed as follow-up was less good at this clinic, and many sample series were incomplete.

An in–house ELISA was developed to measure urine albumin concentrations, using rabbit anti-human albumin polyclonal antibodies (Dako, Denmark) and a human serum albumin standard (Sigma, USA). Full details of the ELISA methodology are presented in the supplementary annex (File S1). One aliquot of each urine sample was sent to a commercial clinical laboratory for measurement of urine creatinine using the Jaffe method. The UACR is presented here as mg-albumin per g-creatinine, with the cutoff defining microalbuminuria taken as ≥30 mg/g [17]. The UACR measured at the follow-up visit was considered as the baseline for that individual.

### Statistical Analysis

Since there is no data about the diagnostic and/or prognostic potential of UACR values in dengue no formal sample size could be calculated. All evaluable patients enrolled in the community cohort study were included in the analysis. Continuous variables were described as median and inter-quartile ranges (IQR) while categorical variables were summarized as frequency and percentage. UACR values were log transformed prior to all analyses to normalize their distribution. The association of baseline UACR values with age, weight, and sex, was assessed with a multivariable linear regression model. Similarly, associations between UACR at presentation and other laboratory features were assessed with linear regression models.

The diagnostic utility of enrolment UACR in differentiating between dengue and OFI was assessed using univariate and multivariable logistic regression and compared to other enrolment clinical and laboratory features (headache, muscle pain, fatigue, chills, vomiting, abdominal pain, diarrhea, skin bleeding, mucosal bleeding, cough, platelet count, hematocrit and white blood cell count) in terms of the odds ratio and the area under the receiver operating characteristic curve (AUC) for continuous features or sensitivity and specificity for binary features.

In patients with confirmed dengue, we assessed the association of enrolment UACR and the enrolment clinical and laboratory measures noted above with the following outcome measures: the platelet nadir; overall percentage haemoconcentration; the development of any of the WHO warnings signs - severe abdominal pain, persistent vomiting, mucosal bleeding, increasing haemoconcentration with a falling platelet count (here defined as haemoconcentration ≥15% with a platelet count ≤50,000/mm^3^); and requirement for hospitalization. Analyses were based on linear regression for continuous outcomes and logistic regression for binary outcomes.

All regression analyses examining the association of enrolment UACR with other measurements were adjusted for the following potential confounding factors: age, sex, day of illness at enrolment and study site. Potential differences in the usefulness of enrolment UACR for diagnosis and risk prediction by study site were assessed with likelihood-ratio tests for interactions.

The evolution of UACR over time in the OFI and confirmed dengue patients, respectively, were investigated using a linear mixed effects model which modeled the mean time trend of UACR as a natural cubic spline function with 4 degrees of freedom adjusted for age and sex, and included a random intercept and slope.

All analyses were performed with R version 2.13.2 [18]. Mixed effects models were fitted with the companion R package lme4. [19].

## Results

### Characteristics of the Study Population

**Figure 1:**
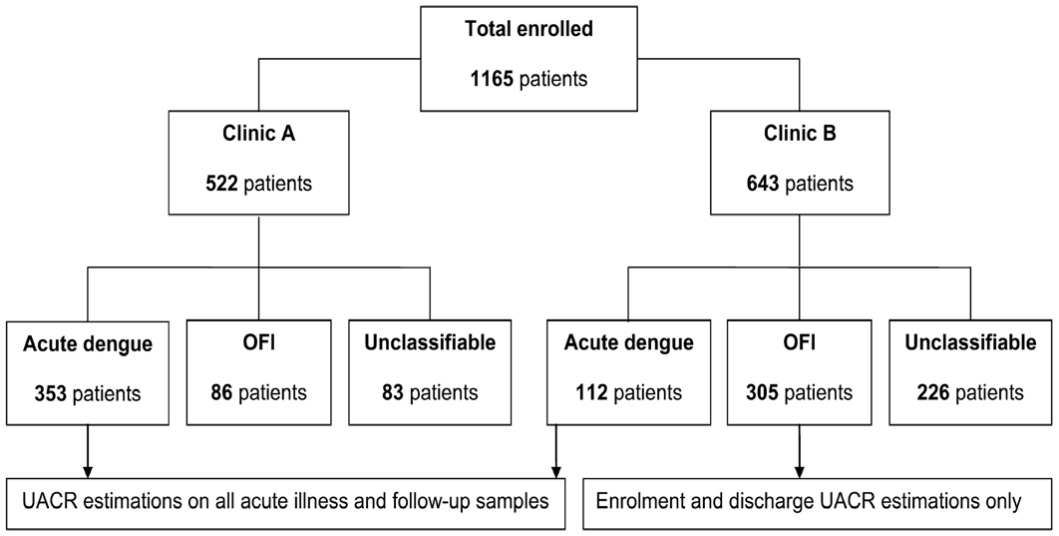
Number of patients enrolled to the study at the two community clinics, according to final diagnosis.

Between October 2005 and December 2008, 1165 children were recruited at the two study sites, of whom 465 (40%) patients had confirmed dengue (353 patients from Clinic A and 112 patients from Clinic B), and 391 (34%) patients were diagnosed with OFI (86 patients from Clinic A and 305 patients from Clinic B). The remaining 309 (27%) children were unclassifiable, in general because of poor clinic attendance limiting the samples available for convalescent serology. This report focuses only on the confirmed dengue and OFI patient groups (Figure 1).

The main demographic and clinical characteristics of the study populations are summarized in Table 1. Some differences were apparent in the populations attending the two clinics. The proportion of confirmed dengue patients was higher at Clinic A and these patients tended to be older and to present later than the dengue patients seen at Clinic B. Characteristics such as the enrolment platelet and white blood counts, overall platelet nadir and degree of haemoconcentration indicate that the patients presenting to Clinic A were also more severe than their counterparts presenting to Clinic B. All the patients who developed DSS came from Clinic A although hospitalization for observation was more common from Clinic B. The pattern of serotypes identified in the confirmed dengue patients at the two sites also differed, with more DENV-2 and less DENV-3 found at Clinic B, but the number of these isolates was relatively small.

**Table 1:** Clinical and laboratory characteristics for the confirmed dengue and OFI patients groups.

	Clinic A (N = 439)		Clinic B (N = 417)		
	Acute dengue	OFI	Acute dengue		OFI
	(N = 353)	(N = 86)	(N = 112)		(N = 305)
**Demographic characteristics**					
**Sex** – Male	188 (53)	47 (55)	62 (55)		169 (55)
**Age** (years)	12 (9–14)	10 (8–12)	10 (9–12)		9 (8–11)
**Weight** (kg)	36 (28–43)	30 (25–39)	30 (25–36)		27 (22–33)
**Day of illness at enrolment**					
1	58 (16)	7 (8)	20 (18)		65 (21)
2	145 (41)	44 (51)	87 (78)		230 (75)
3	111 (31)	31 (36)	5 (5)		9 (3)
4	39 (11)	4 (4)	0 (0)		1 (0)
**Dengue serotypes identified**					
DENV1	217 (61)	NA	59 (53)		NA
DENV2	47 (13)	NA	35 (31)		NA
DENV3	76 (22)	NA	14 (12)		NA
DENV4	3 (1)	NA	1 (1)		NA
Unknown	10 (3)	NA	3 (3)		NA
**Clinical and laboratory features present at enrolment**					
**Headache**	322 (91)	71 (83)	92 (82)	234 (77)	
**Muscle pain**	253 (72)	31 (36)	10 (9)	25 (8)^a^	
**Fatigue**	298 (84)	70 (81)	56 (50)	123 (40)	
**Chills**	184 (52)	30 (35)	61 (54)	162 (53)	
**Vomiting**	250 (71)	51 (59)	36 (32)	50 (16)	
**Abdominal pain**	89 (25)	13 (15)	9 (8)	39 (13)^a^	
**Diarrhoea**	23 (7)	7 (8)	0 (0)	11 (4)	
**Skin bleeding**	13 (4)	0 (0)	7 (6)	4 (1)	
**Mucosal bleeding**	12 (3)	0 (0)	1 (1)	3 (1)	
**Cough**	22 (6)	25 (29)	29 (26)	129 (42)	
**Platelet count** (x1,000 cells per mm^3^)	183^c^ (143–222)	218^a^ (177–256)	236^a^ (192–264)	263^a^ (221–298)	
**Hematocrit** (%)	38.8^c^ (36.6–41.3)	38.4^a^ (36.2–41.4)	39.1^a^ (36.1–40.6)	38.9^a^ (36.7–41.2)	
**WBC** (x1,000 cells per mm^3^)	3.8^e^ (2.8–5.5)	5.9^b^ (4.1–7.9)	6.1^d^ (4.7–7.7)	8.3^f^ (6.0–11.4)	
**Summary of the subsequent course of illness and any complications noted**					
**Mucosal bleeding**	14 (4)	2 (2)	5 (4)		4 (1)
**Persistent vomiting** *	9 (3)	0	6 (5)		2 (1)
**Severe abdominal pain** *	19 (5)	1 (1)	0		1 (0)
**Dengue shock syndrome (DSS)**	6 (2)	0	0		0
**Admission to hospital**	10 (3)	0	21 (19)		1 (0)
**Days of illness at defervescence**	5 (5–6)	5 (4–6)	4 (3–5)		3 (3–4)
**Platelet nadir** (x1,000 cells per mm^3^)	96 (69–134)	195 (156–221)	140 (106–195)		223 (186–265)
**Overall percentage hemoconcentration** (%)	11.7^a^ (5.6–19.4)	7.8 (3.8–14.8)	7.3^a^ (2.4–15.8)		5.4 (0.3–10.9)

Continuous variables are summarized as median (interquartile range); categorical variables are summarized as frequency (%).

Missing values for:^ (a)^ up to 3, ^(b)^ 5, ^(c)^ 9, ^(d)^ 12, ^(e)^ 20, ^(f)^ 48 cases.

OFI: other febrile illness.

* If present, the severity of clinical symptoms was evaluated each day by study physicians using a pre-defined three point scale. For this analysis participants were considered to have persistent vomiting or severe abdominal pain if they scored two or more on the relevant scale, on any day during the acute illness.

### Baseline (Follow-up) Urine Albumin Creatinine Ratios

A total of 614 patients attended the final follow-up visit, of whom 356 (58%) had confirmed dengue and 258 (42%) had OFI (Table 2). All patients had fully recovered clinically by this visit and there were 429 urine samples available for UACR estimation. The median (IQR) UACR measured in all follow-up samples was 6.1 (2.2–13.8) mg/g creatinine, and 53/429 (12%) children had microalbuminuria: 44/356 (12%) with recent dengue and 9/73 (12%) with recent OFI. Baseline UACRs of patients from Clinic A (median (IQR) of 5.8 (2.4–12.2) and 5.8 (1.7–13.6) mg/g creatinine for dengue and OFI patients respectively) were lower than patients from Clinic B (10.5 (4.0–19.6) mg/g creatinine) but this difference was not statistically significant (t-test of log-values: p = 0.13). A multivariable linear regression with sex, age and weight as covariates (factors previously shown to be associated with UACR in other studies) confirmed a relationship for both sex and age with baseline UACR in this population [20]–[22]. Specifically, the model predicted a 2.17 (95%CI: 1.63–2.90, p<0.0001) times higher UACR in females and a 1.12 (95%CI: 1.05–1.20, p = 0.001) times higher UACR per +1 year increase in age, whereas weight was not significantly associated with UACR (p = 0.07).

**Table 2:** Urine albumin creatinine ratio (UACR) values.

	Clinic A (N = 439)		Clinic B (N = 417)		Total (N = 856)	
	Acute dengue	OFI	Acute dengue	OFI	Acute dengue	OFI
	(N = 353)	(N = 86)	(N = 112)	(N = 305)	(N = 465)	(N = 391)
**Attended final follow-up visit**	298	73	58	185	356	258
	(84)	(85)	(52)	(61)	(77)	(65)
**Baseline UACR–mg/g creatinine**	5.8	5.8	10.5	NA	6.1	NA
(for those attending final follow-up)	(2.4–12.2)	(1.7–13.6)	(4.0–19.6)		(2.5–13.8)	
**UACR at enrolment**	16.0	16.3	18.2^a^	13.4^a^	16.5^a^	13.6^a^
(mg/g creatinine)	(8.0–30.5)	(5.6–29.2)	(9.2–46.6)	(5.7–25.4)	(8.6–33.2)	(5.6–25.9)
**Microalbuminuria at enrolment**	88	22	36^a^	60^a^	124^a^	82^a^
(≥30 mg/g creatinine)	(25)	(26)	(33)	(20)	(27)	(21)
**Peak UACR** b	41.3	37.0	39.6	NA	41.0	NA
(mg/g creatinine)	(22.3–107.1)	(17.6–83.6)	(24.6–99.5)		(22.7–103.9)	
**Microalbuminuria ever present** c	222	50	72	NA	294	NA
(≥30 mg/g creatinine)	(63)	(58)	(64)		(63)	
**Day of illness of peak UACR**	5	4	3	NA	5	NA
	(3–6)	(3–5)	(2–5)		(3–6)	

Continuous variables are summarized as median (interquartile range); categorical variables are summarized as frequency (%).

For Clinic A, both dengue and OFI patients had urine albumin quantification performed on all urine samples, while for Clinic B urine albumin quantification was performed on the serial urine samples from confirmed dengue patients only, plus the enrolment and discharge day urine samples from the OFI patient group.

a Missing values for 3 patients in each group.

b The maximum UACR value observed during the acute illness.

c Peak UACR ≥30 mg/g creatinine at any time during the acute illness.

### Urine Albumin Creatinine Ratios in the Acute Illness

As presented in Table 2, UACRs at study enrolment were increased compared to the corresponding baseline values for both the dengue and OFI groups (median (IQR) at enrolment of 16.5 (8.6–33.2) mg/g creatinine for dengue and 13.6 (5.6–25.9) mg/g creatinine for OFI, both clinics combined). In the dengue group, we found a significant negative correlation between enrolment UACR and enrolment platelet count – specifically, UACR values were increased by 2% for each 10,000 cells/mm^3^ reduction in platelet count (p = 0.03, linear regression adjusted for age, sex, day of illness at enrolment and study site). We found no significant associations between enrolment UACR and haematocrit (p = 0.64) or white blood cell count (p = 0.35).

**Figure 2:**
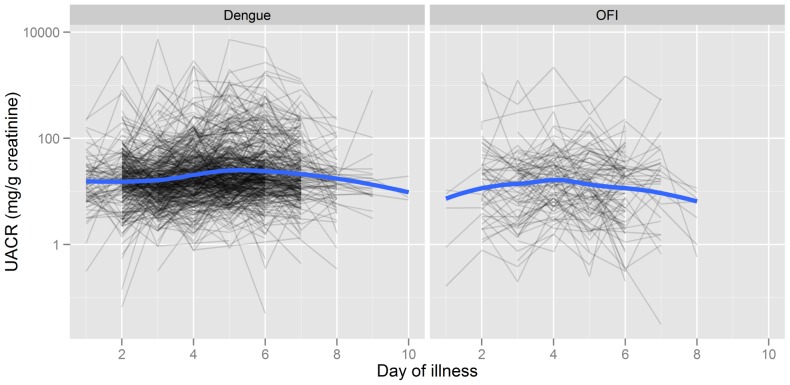
Longitudinal dynamics of UACRs in the confirmed dengue and OFI patient groups. All patients from Clinic A, and the confirmed dengue patients from Clinic B, are included. A significant time trend was observed in the dengue patients (p<0.0001), peaking on day 5 of illness, but no trend was apparent in the OFI patients (p = 0.22). The grey lines represent the evolution of the UACRs over time for each patient. The blue lines correspond to loess scatter plot smoothers.

Longitudinal analysis of patterns in UACR evolution during the acute illness found a significant time trend for dengue patients (p<0.0001) but not for the OFI group (p = 0.22). However, examining individual UACR trajectories revealed considerable within-patient variability making it difficult to identify common patterns over time. As shown in Figure 2 and Table 2 dengue patients generally had higher UACR values than OFI patients although a similar proportion of patients in each group had evidence of microalbuminuria at some time during the acute illness. Peak UACR values for the dengue patients were observed at a median (IQR) day 5 (3–6) of illness.

### Enrolment Urine Albumin Creatinine Ratio and Diagnosis of Dengue

Enrolment UACR was significantly associated with a dengue diagnosis in both univariate and multivariable analyses and there was no evidence that the effect of UACR on dengue diagnosis differed by clinic (Table 3). However, the size of the UACR effect was small and the discrimination between OFI and dengue patients based on UACR (AUC = 0.56, 95% CI = 0.52–0.59) alone was weaker than for enrolment WBC (AUC = 0.79, 95% CI = 0.76–0.82) or platelet count (AUC = 0.74, 95% CI = 0.70–0.77) alone. Also, the presence of microalbuminuria was not a clear indication for a dengue diagnosis (odds ratio = 1.44, 95% CI 0.98–2.11, p = 0.06), and although microalbuminuria had fair specificity (79%), its sensitivity was low (27%).

**Table 3:** Univariate and multivariable logistic regression analysis examining relationships between clinical and laboratory features present at enrolment and diagnosis of dengue in the study cohort of 856 patients.

	Univariate OR(95%CI)	UnivariateP value	AUC or Sensitivity/Specificity (%)	Multivariable OR^e^(95% CI)	MultivariableP value
**UACR ^a^**	1.13 (1.03–1.25)	0.008	0.56	1.15 (1.03–1.29)	0.02
**(per 2-fold increase)**					
**Platelet count ^b^**	0.92 (0.90–0.95)	<0.0001	0.74	0.97 (0.93–1.00)	0.07
**(+10,000 cells/mm^3^)**					
**Hematocrit ^c^**	0.96 (0.91–1.01)	0.10	0.51	0.96 (0.91–1.02)	0.18
**(+1%)**					
**White blood cell count ^d^**	0.77 (0.72–0.83)	<0.0001	0.79	0.76 (0.70–0.82)	<0.0001
**(+1000 cells/mm^3^)**					
**Microalbuminuria^a^**	1.44 (0.98–2.11)	0.06	27/79	NA	NA
**Headache**	1.51 (0.96–2.38)	0.07	89/22	1.00 (0.57–1.75)	0.99
**Muscle pain**	2.74 (1.83–4.11)	<0.0001	57/86	2.47 (1.47–4.16)	0.0006
**Fatigue**	1.29 (0.90–1.85)	0.17	76/51	1.10 (0.67–1.81)	0.70
**Chills**	1.32 (0.95–1.84)	0.10	53/51	1.14 (0.73–1.79)	0.56
**Vomiting**	2.24(1.55–3.21)	<0.0001	62/74	2.78 (1.75–4.43)	<0.0001
**Abdominal pain**	1.25 (0.80–1.97)	0.32	21/87	1.45 (0.80–2.65)	0.23
**Diarrhea**	0.52 (0.25–1.11)	0.09	5/95	0.41 (0.15–1.15)	0.08
**Skin bleeding**	4.92 (1.64–18.24)	0.004	4/99	4.49 (1.01–25.45)	0.05
**Mucosal bleeding**	2.13 (0.58–10.52)	0.27	3/99	0.79 (0.13–15.37)	0.83
**Absence of cough**	3.31 (2.22–4.98)	<0.0001	89/40	3.83 (2.38–6.23)	<0.0001

Logistic regression models were adjusted for age, sex, day of illness at enrolment and study site (Clinic A vs Clinic B). Interaction tests revealed no evidence for a difference in the effect of UACR by study site (p = 0.13 for univariate analysis and 0.27 for multivariable analysis, likelihood ratio test). AUC and sensitivity/specificity were calculated without adjustment.

All factors from the univariate analysis were included in the multivariable model apart from microalbuminuria, since this was already represented by the UACR value.

There were ^(a)^ 6, ^(b)^ 15, ^(c)^ 16 and ^(d)^ 85 cases with missing values for these parameters.

(e) A total of 752 patients with complete covariate information were included in the multivariable model.

OR: odds ratio.

AUC: area under the receiver operating characteristic curve.

We also found significant associations for muscle pain, vomiting, and absence of cough at enrolment, with confirmed dengue, but with variable sensitivities and specificities (Table 3). Skin bleeding, although infrequent, was associated with a diagnosis of dengue. In the multivariable models, in addition to enrolment UACR only white blood count, muscle pain, vomiting, and absence of cough were independently associated with confirmed dengue.

### Enrolment Urine Albumin Creatinine Ratio as a Prognostic Factor

#### Platelet nadir

Using data from the two clinics combined, both univariate and multivariable linear regression analysis showed no significant association between enrolment UACR and the platelet nadir in the confirmed-dengue patients (p = 0.12 and 0.74 respectively, Table 4). Also the presence of microalbuminuria at enrolment was not clearly associated with a lower platelet nadir (p = 0.08), although associations were demonstrated for a number of other enrolment characteristics, including the platelet and white blood counts, muscle pain, chills, vomiting, and abdominal pain. However, multivariable analysis indicated a significant effect for enrolment platelet count only.

**Table 4:** Univariate and multivariable linear regression analysis examining relationships between clinical and laboratory features present at enrolment and the platelet nadir in 465 dengue patients.

	Univariate Effect (95%CI)(×1,000 cells/mm^3^)	UnivariateP value	Multivariable Effect ^d^ (95% CI)(×1,000 cells/mm^3^)	MultivariableP value
**UACR^ a^**	−2.2 (−4.9, 0.6)	0.12	0.4 (−2.0, 2.8)	0.74
**(per 2-fold increase)**				
**Platelet count^ b^**	4.8 (4.1, 5.6)	<0.0001	4.8 (4.0, 5.6)	<0.0001
**(+10,000 cells/mm^3^)**				
**Hematocrit ^b^**	−1.0 (−2.4, 0.4)	0.16	−0.3 (−1.5, 0.9)	0.62
**(+1%)**				
**White blood cell count ^c^**	2.7 (0.5, 4.8)	0.02	−1.6 (−3.7, 0.5)	0.13
**(+1,000 cells/mm^3^)**				
**Microalbuminuria**	−9.6 (−20.2, 1.1)	0.08	NA	NA
**Headache**	−12.8 (−28.2, 2.5)	0.10	−14.8 (−30.0, 0.5)	0.06
**Muscle pain**	−12.4 (−23.6, −1.1)	0.03	0.4 (−11.1, 12.0)	0.94
**Fatigue**	−11.2 (−23.0, 0.6)	0.06	−4.7 (−16.5, 7.1)	0.43
**Chills**	−11.9 (−21.2, −2.6)	0.01	−6.2 (−15.2, 2.9)	0.18
**Vomiting**	−17.4 (−27.7, −7.1)	0.001	−5.7 (−15.5, 4.0)	0.25
**Abdominal pain**	−20.9 (−32.9, −8.9)	0.0007	−10.6 (−21.6, 0.5)	0.06
**Diarrhea**	−16.3 (−38.0, 5.4)	0.14	0.4 (−19.3, 20.0)	0.97
**Skin bleeding**	−4.2 (−27.4, 19.1)	0.72	2.1 (−19.1, 23.3)	0.85
**Mucosal bleeding**	−12.5 (−41.0, 16.0)	0.39	−5.9 (−31.3, 19.5)	0.65
**Absence of cough**	13.4 (−2.0, 28.9)	0.09	12.9 (−0.9, 26.6)	0.07

The linear regression models were adjusted for age, sex, day of illness at enrolment and study site. There was no evidence for an interaction between UACR and study site (p = 0.91 for univariate analysis and 0.59 for multivariable analysis).

All factors from the univariate analysis were included in the multivariable model apart from microalbuminuria, since this was already represented by the UACR value.

There were ^(a)^ 3, ^(b)^ 11 and ^(c)^ 32 cases with missing values for these parameters.

(d) A total of 423 patients were included in the multivariable model.

#### Overall haemoconcentration

We found no evidence of a relationship between enrolment UACR and overall percentage haemoconcentration in either the univariate or the multivariable linear regression analyses (p = 0.37 and 0.46 respectively). We found associations in the univariate analysis between abdominal pain and mucosal bleeding with overall percentage hemoconcentration, but no significant associations were found in the multivariable analysis.

#### Warnings signs and hospitalization

Among all 465 confirmed-dengue patients 73 (16%) developed one or more of the WHO warning signs and 31 (7%) were hospitalized (6 with DSS; the others for close observation). We found no associations between enrolment UACR and either development of warning signs or need for hospitalization (both p>0.1, univariate logistic regression).

## Discussion

Development of plasma leakage, potentially resulting in hypovolaemic shock, is the most important complication associated with dengue infections. Here we describe the magnitude and kinetics of urinary albumin excretion, expressed in terms of UACR measurements, in more than 800 Vietnamese children with suspected dengue, aiming to assess the usefulness of this marker as a diagnostic tool and as an early marker for development of clinically important plasma leakage. Although UACRs were increased in the confirmed dengue patients, with a significant time trend showing peak values around the critical period for dengue-associated plasma leakage, urine albumin excretion was also increased in the OFI comparison group. Although the dengue patients generally had higher UACRs than the OFI patients, discrimination between the two diagnostic groups in the early febrile phase was poor. Secondly UACRs did not prove useful in predicting either development of warning signs for severe dengue or need for hospitalization.

The follow-up values for the Vietnamese children, here taken as representing the baseline state, were generally similar to the UACRs reported previously for healthy children, and the relationships with gender and age followed established patterns [20]–[22]. However the proportion of subjects categorised as having microalbuminuria at this time was quite high (12%), using the conventional definition of a UACR value above 30 mg/g creatinine [22], [23]. Although racial differences could contribute to this finding [22], it may be that the timing of the follow-up visit 2–4 weeks after the acute illness influenced the results. Absolute values for most of the patients with microalbuminuria were only marginally elevated (only 2 patients had values above 300 mg/g creatinine, the threshold for macroalbuminuria), and although the study participants had recovered clinically by this time minor residual endothelial dysfunction may have contributed to the persisting abnormalities.

Marked proteinuria has been demonstrated previously in association with dengue shock syndrome [12], but this study shows that low level albuminuria is extremely common, even in relatively mild dengue infections, and occurs throughout the evolution of the illness. The magnitude of the changes observed in the confirmed dengue cases was only marginally greater than among the OFI group, most of whom are likely to have had other viral infections. Increased UACRs have been reported in many conditions including cardiovascular disease, critical illness, sepsis syndromes and autoimmune disorders [7], [24]–[26], with a variety of cytokines, inflammatory mediators and other immune effector mechanisms postulated to alter endothelial function thereby resulting in increased vascular permeability [27]–[29]. Whilst it was not possible to differentiate a generalized increase in permeability from a localized renal effect in this study, the reports of increased UACRs across a wide variety of systemic disorders, in many cases independent of renal function, indicate that generalized microvascular dysfunction can be associated with albuminuria; our findings demonstrate that albuminuria is a common, though usually minor, feature of many childhood febrile illnesses.

With respect to diagnosis of dengue, in addition to the small effect of enrolment UACR we also found independent associations for muscle pain, vomiting, absence of cough, and a lower white count at enrolment, but the specificity/sensitivity or AUC of these clinical and laboratory features was generally poor. Only a small number of the dengue patients developed DSS, and the performance of the enrolment UACR with respect to the other outcomes assessed was disappointing. Several other groups have examined relationships between early clinical and laboratory features and either diagnosis or prognosis of dengue infections [5], [6], [30]. Although a range of parameters have been identified, as yet there is no consensus on the most useful combination, and the experience of many clinicians is that dengue is typically non-specific in nature until or unless complications develop. Further work using very large datasets is needed to determine whether simple clinical/laboratory diagnostic or warning signs can indeed be identified. Given the rapidly evolving nature of the illness, especially apparent in haematological parameters such as the platelet and white blood counts, it is possible that changes in repeated measures may be more powerful in this regard than single time-point information.

Several other factors may have contributed to the limited diagnostic and prognostic utility of early UACR assessments that we demonstrated. While interaction tests found no evidence that the diagnostic or prognostic utility of UACR differed by clinic, we acknowledge the clear differences between the patient populations recruited at the two study sites as a limitation of our study. Clinic A recruited mainly dengue cases, while Clinic B recruited a larger number of OFI cases and was less successful at organizing the daily follow-up and final convalescent visits. Also the dengue patients enrolled at clinic A were both more likely to develop shock and to be severe in terms of the summary measures that we assessed, compared to their counterparts at Clinic B. A possible explanation for these differences could be that Clinic A operates in the evenings rather than during the day (i.e. out of school hours) and patients attending this clinic were generally older and thus more likely a) to have dengue than OFI, and b) to have secondary rather than primary dengue infections. Unfortunately however we were unable to compare immune status between the two dengue-infected patient groups due to the lack of suitable convalescent samples from a large proportion of Clinic B cases. Finally, the timing of urine collection (usually in the morning at Clinic A) is likely to have resulted in slightly higher UACR values in the OFI group who were primarily enrolled at Clinic A, compared to the evening measurements in most of the dengue patients [31], [32].

Another point to consider is the marked variability in serial UACR measurements both within and between individual patients. Urine albumin excretion over 12 or 24 hrs is usually taken as the gold standard for assessment of albuminuria, but measurement of the UACR on spot urine samples is more practical and is generally considered an acceptable surrogate measure [33]. Although the UACR technique is intended to adjust for fluctuations in fluid intake and hydration status, considerable variability within individuals is recognized [34], [35]. Given the complex fluid balance status in patients with increased vascular permeability the method may not be appropriate for use in suspected dengue cases. However, it is clear that similar variability was seen in both the OFI and the dengue patient groups suggesting that the problem is intrinsic to the UACR method and that comparisons between the disease groups remain valid. Although significant differences in urine albumin excretion might have been detected using timed urine collections, it would not be feasible to apply this technique to large-scale community based assessments of children with suspected dengue.

In conclusion, although we have demonstrated slightly increased UACRs throughout the evolution of dengue infection in Vietnamese children, the marked within-patient variability and limited discrimination from the OFI group suggest that the point-of care tests that are available to measure UACR would not be sufficiently robust to be useful for early diagnosis or risk prediction in dengue endemic areas.

## Supporting Information

File S1 **Supplementary materials and methods.**
(DOCX)
