# Supplementary material for: Assessment of Microalbuminuria for Early Diagnosis and Risk Prediction in Dengue Infections
Source: PLoS One. 2013 Jan 22;8(1):e54538. doi: 10.1371/journal.pone.0054538 (PMC3551767; doi:10.1371/journal.pone.0054538)
Supplement: File S1 — Supplementary materials and methods. (DOCX) [file pone.0054538.s001.docx]

**File S1: Supplementary materials and methods**

**Quantitative urine albumin ELISA**

A standard sandwich ELISA format was employed using the following reagents:

1. Rabbit anti-human albumin polyclonal antibody (Dako, Denmark)

2. 1% NHS-LC-LC-biotin (Thermo Fisher Scientific Inc, USA)

3. Biotinylated rabbit anti-human albumin polyclonal antibody – synthesis described below

4. Sodium bicarbonate, pH 9.6 (Sigma, USA)

5. Skim milk (Sigma, USA)

6. Human serum albumin (Sigma, USA)

7. Streptavidin horseradish peroxidase (Dako, Denmark)

8. o-Phenylenediamine dihydrochloride (OPD)/Urea H_2_O_2_ buffer (Sigma, USA)

**Synthesis of biotinylated anti-human albumin polyclonal antibody**

A solution of 10mg/ml rabbit anti-human albumin polyclonal antibody was first mixed well in 100 mM bicarbonate solution at pH 8.5 with 1% NHS-LC-LC-biotin in DMSO, at a ratio of 50:1, and then incubated on ice for 3 hours. The product was purified and condensed using a Microcon YM30 microconcentrator (Millipore Corp., USA) to obtain a final concentration of the biotinylated antibody of approximately 2 mg/ml.

**Quantitative urine albumin ELISA**

96 well-plates (Maxisorp, Nunc, USA) were initially coated with rabbit anti-human albumin polyclonal antibody at a dilution of 1/5000 in sodium bicarbonate, pH 9.6, and incubated overnight at 4^o^C, then blocked with 2% skim milk. Subsequently, the plates were incubated for 1 hour at room temperature with 4 serial dilutions of the urine samples ranging from 1/200 to 1/25000, and the human albumin standard solutions ranging from 0.3 – 10 ng/ml. Captured albumin was detected with the biotinylated antibody at a 1/10,000 dilution, and the reaction was developed with streptavidin horseradish peroxidase and OPD/urea H_2_O_2_ buffer and then stopped with 10% H_2_SO_4_. The optical density was measured at a wavelength of 490 nm using a Microplate reader and Microplate Manager software (Biorad, USA). All samples were run in duplicate to give average final results with CVs less than 7%. The limit of detection of this assay was 0.3 ng/ml.
